# Cachexia, chorea, and pain in chronic nonbacterial osteitis and inflammatory bowel disease: a case report

**DOI:** 10.1186/s13256-023-03894-1

**Published:** 2023-05-31

**Authors:** Ladan Agharokh, Katherine Mamola, Andrew G. Yu, Annette L. Medina, Bhaskar Gurram, Julie Fuller, Jason Y. Park, Weina Chen, Veena Rajaram, Matthew R. Hammer, Jeff L. Waugh

**Affiliations:** 1 Division of Pediatric Hospital Medicine, Department of Pediatrics, University of Texas Health Science Center at Houston, 6431 Fannin St., Suite JJL 210-D, Houston, TX 77030 USA; 2 Division of Pediatric Hospital Medicine, Department of Pediatrics, University of Texas-Southwestern Medical Center, Dallas, TX USA; 3 Division of Pediatric Gastroenterology, Department of Pediatrics, Nicklaus Children’s Hospital, Miami, FL USA; 4 Division of Pediatric Gastroenterology, Department of Pediatrics, University of Texas-Southwestern Medical Center, Dallas, TX USA; 5 Division of Pediatric Rheumatology, Department of Pediatrics, University of Texas-Southwestern Medical Center, Dallas, TX USA; 6 Department of Pathology, University of Texas-Southwestern Medical Center, Dallas, TX USA; 7 Division of Pediatric Radiology, Department of Radiology, University of Texas-Southwestern Medical Center, Dallas, TX USA; 8 Division of Child Neurology, Department of Pediatrics, University of Texas-Southwestern Medical Center, Dallas, TX USA

**Keywords:** Inflammatory bowel disease, Chronic nonbacterial osteomyelitis, Chorea, Extraintestinal manifestations

## Abstract

**Background:**

Inflammatory bowel disease is an inflammatory disorder that primarily impacts the gastrointestinal tract, leading to malnutrition and chronic microscopic intestinal blood loss. Uncontrolled systemic inflammation can impact other parts of the body, known as extraintestinal manifestations. Up to 25% of patients with inflammatory bowel disease are reported to have these complications in their skin, joints, bones, eyes, liver, lung, and pancreas (Rogler *et al.* in Gastroenterology 161(4):1118–1132, 2021). Neurologic involvement as extraintestinal manifestations are less common, reported at 3–19%, including neuropathies, demyelination, and cerebrovascular events (Morís in World J Gastroenterol. 20(5):1228–1237, 2014).

**Case presentation:**

A 13-year-old Caucasian boy presented with 1 month of progressive lower-extremity pain, weakness, and weight loss. His physical examination was notable for cachexia, lower-extremity weakness, and chorea. Labs revealed normocytic anemia and systemic inflammation. Imaging revealed symmetric abnormal marrow signal in the pelvis and upper femurs. Pathologic examination of the bone revealed chronic inflammation consistent with chronic nonbacterial osteitis. Endoscopy revealed colonic inflammation consistent with inflammatory bowel disease.

**Conclusions:**

Children and adolescents with musculoskeletal pain lasting more than 2 weeks with systemic signs or symptoms like weight loss should prompt evaluation for systemic inflammatory disorders such as chronic nonbacterial osteitis, which can occur in isolation or associated with inflammatory bowel disease. This patient also had a nonspecific neurologic abnormality, chorea, which resolved with treatment of underlying inflammatory disorder. These extraintestinal manifestations may be concurrent with or precede intestinal inflammation, requiring a high index of suspicion when investigating nonspecific systemic inflammation.

**Supplementary Information:**

The online version contains supplementary material available at 10.1186/s13256-023-03894-1.

## Background

Acute weight loss in children has a broad differential diagnosis, depending on whether there is adequate or inadequate caloric intake. Ongoing weight loss despite adequate caloric intake can be caused by malabsorption, increased caloric demand, or abnormalities in the metabolic pathways that convert food to usable energy. One cause of malabsorptive weight loss, inflammatory bowel disease (IBD), classically presents with weight loss, abdominal pain, and bloody diarrhea. However, this disease can also present atypically without intestinal complaints and only nonspecific symptoms such as weight loss and anemia. Up to 25% of patients who will later be diagnosed with IBD can present with extraintestinal manifestations (EIM): inflammatory disorders of various other organ systems, including the skin, musculoskeletal system, lung, liver, and pancreas. Even more rarely, neurologic disorders such as neuropathies, demyelinating disorders, and cerebrovascular events have been reported as EIM associated with IBD [1–5]. Because EIM can precede the diagnosis of IBD by months to years, these conditions are diagnostically challenging and require a high index of suspicion.

## Case presentation

A 13-year-old Caucasian boy with well-controlled attention-deficit/hyperactivity disorder presented with 1 month of progressive bilateral lower-extremity pain and weakness. He had difficulty standing from a sitting or crouching position due to bilateral buttock and leg pain. He described his pain as sharp, radiating through the buttocks in a sciatic pattern. He denied abdominal pain, constipation, diarrhea, or hematochezia. He had no bowel or bladder incontinence or retention and denied numbness or paresthesias. Associated symptoms included fatigue and an 8-pound weight loss (3.6 kg, then at the 4th percentile, *Z* = −1.74) without fever or a change in appetite.

On examination, he was afebrile with normal vital signs. He appeared cachectic with reduced muscle bulk. He had no rash, palpable lymph nodes, joint swelling, or oral ulcerations; he had no murmur on cardiac examination. Bilateral hip and knee strength was reduced (4/5, both flexion and extension), though distal lower extremity strength was preserved. Pain was exacerbated with both passive and active movement of his hips. When walking, his gait was waddling, antalgic, and wide-based. He had preserved reflexes and sensation to light touch and proprioception throughout.

Initial testing was notable for an elevated C-reactive protein (CRP, 4.0 mg/dL; normal 0–1 mg/dL), markedly elevated erythrocyte sedimentation rate (ESR, 78 mm/hour; normal, 0–15 mm/hour), normocytic anemia (hemoglobin 11.6 g/dL; normal, 12.4–16.3 g/dL), and hypoalbuminemia (3.1 g/dL; normal, 3.6–5.1 g/dL). He had normal platelets, electrolytes, liver enzymes, and creatine kinase levels. Magnetic resonance imaging (MRI) of the brain and full spine, electroencephalogram, lumbar puncture (basic indices as well as oligoclonal bands), and electromyogram with nerve conduction study were unremarkable. An abdominal ultrasound was negative for any solid mass, infiltrative tumor, or abscesses. Screening for vitamin deficiencies was negative. His pain and mobility slowly improved with physical and occupational therapy over 4 days, so he was discharged home with close follow-up of his weight and nutritional status.

**Fig. 1:**
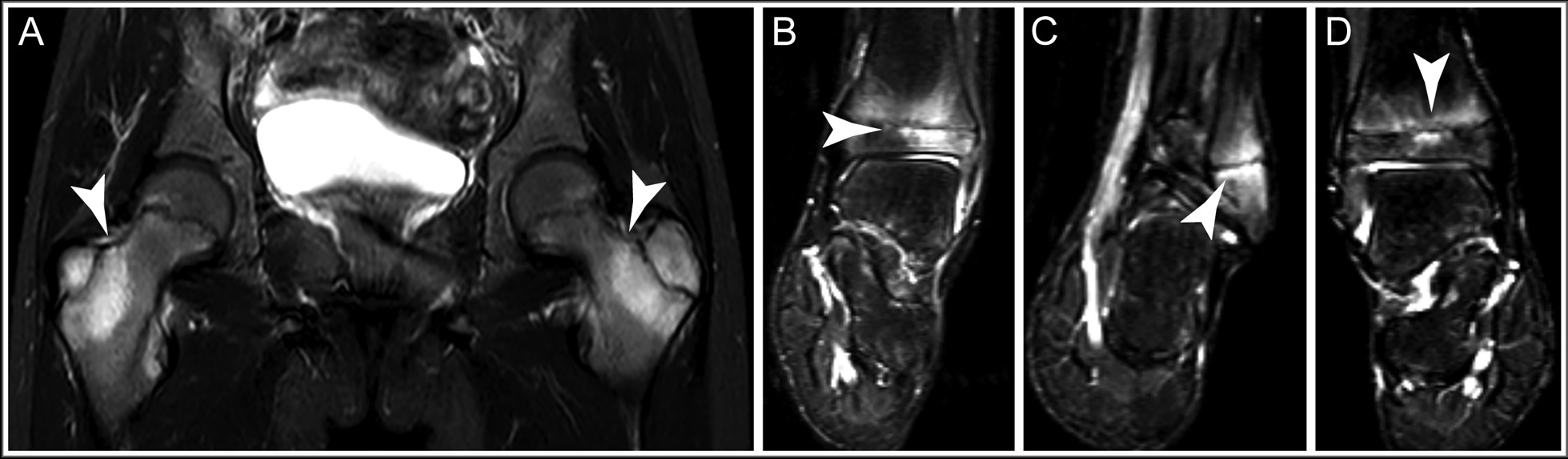
Magnetic resonance imaging of the hips and ankles. Short-tau inversion-recovery (STIR) sequence of the hips and ankles demonstrated widespread osseous inflammation, as indicated by white arrowheads. Bilateral inflammation of the femur (**A**) was evident along the greater trochanter apophyseal plates. In the left tibia (**B**) and fibula (**C**), inflammation likewise paralleled the physes. Inflammation was more widespread (**D**), involving the majority of the metaphysis and epiphysis. Joint effusions with trace volume were evident at each of these sites

Four weeks later, he returned with acutely worsening symptoms of bilateral lower-extremity pain and weakness, and his weight had not changed. He continued to show evidence of systemic inflammation with elevated CRP (peak 4.99 mg/dL; normal, 0–1 mg/dL), elevated ESR (peak 92 mm/hour; normal, 0–15 mm/hour), and normocytic anemia (9.9 g/dL; normal, 12.4–16.3 g/dL). On physical examination, he was noted to be ill-appearing, with cachexia, bilateral lower-extremity proximal weakness to resistance, and a positive Gower sign, as well as low-amplitude chorea evident in the face, the tongue, and the upper extremities more than lower extremities (Additional file 1: Video S1). The focality of his buttock pain (bilateral, 3–4 cm lateral to the lumbosacral junction) suggested the need for MRI of the pelvis, which demonstrated symmetric, bilateral abnormal marrow signal in the medial iliac bones and greater trochanters (Fig. 1).

**Fig. 2:**
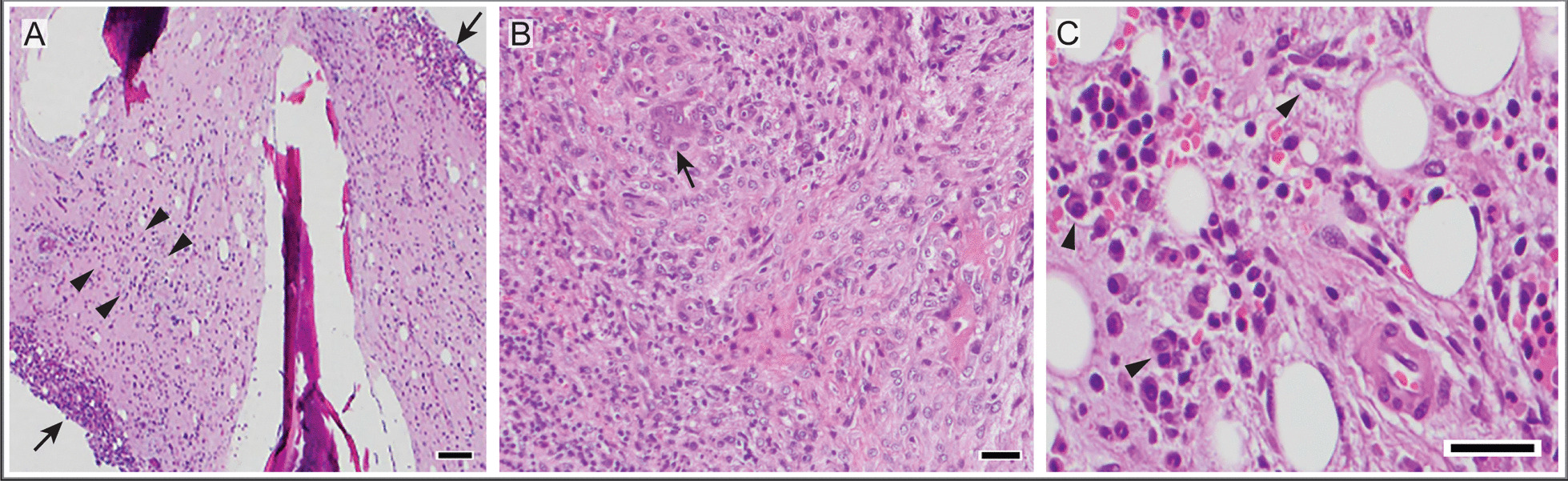
Bone marrow histology. Biopsy of the left and right iliac crest bone marrow revealed histiocytic chronic inflammatory changes, including stromal edema and fibrosis, increased histiocytes (long arrows), polytypic plasma cells (arrowheads), and diminished hematopoiesis. Image from hematoxylin- and eosin-stained tissue (**A**, 100×; **B**, 200×; **C**, 400×)

This marrow infiltrative process prompted whole-body MRI to evaluate for oncologic, infectious, metabolic/toxic, and rheumatologic etiologies. No mass lesions were identified; however, there were multiple foci of abnormal marrow signal in the bilateral distal fibular metaphyses and epiphyses and left tibial epiphysis (Fig. 1), and a subtle abnormality in the proximal left radius. Whole-body positron emission tomography–computed tomography (PET–CT) revealed normal metabolic activity in these areas of abnormal MRI signal. Bone marrow aspirate and biopsy showed evidence of histiocytic chronic inflammatory changes (Fig. 2), though stains were negative for malignancy, including Langerhans cell histiocytosis (LCH) and hemophagocytic lymphohistiocytosis (HLH).

Infectious evaluation included bacterial [cat scratch disease (*Bartonella henselae*) and brucellosis (*Brucella spp*)], mycobacterial (tuberculosis and nontuberculous mycobacteria), fungal (coccidiomycosis, histoplasmosis, and aspergillosis), viral (Epstein–Barr virus, cytomegalovirus, and human immunodeficiency virus), tick-borne (Lyme disease), and parasitic (toxoplasmosis and *Trichinella*) etiologies. Specimens sent for culture and other infectious evaluations, including blood, urine, cerebrospinal fluid, bone marrow biopsy, and bone biopsy from the right femoral lesion were all negative.

The presence of chorea triggered evaluation for toxic and metabolic etiologies, as well as various systemic autoimmune diseases, such as acute rheumatic fever (ARF) and systemic lupus erythematosus. Electrocardiogram (ECG) showed a normal PR interval, and echocardiogram was negative for valvular disease that would suggest ARF. Comprehensive urine drug profile and serum heavy metals screen were negative. Evaluation for Wilson’s disease was negative (normal ceruloplasmin level). Review of his prior brain MRI confirmed the absence of atrophy or focal lesions associated with chorea.

A 3-day calorie count showed intake of 150% of his caloric needs with concurrent weight loss. He underwent endocrine evaluation for increased catabolic states (for example, hyperthyroidism or Addison disease), but thyroid function and cortisol levels were normal.

**Fig. 3:**
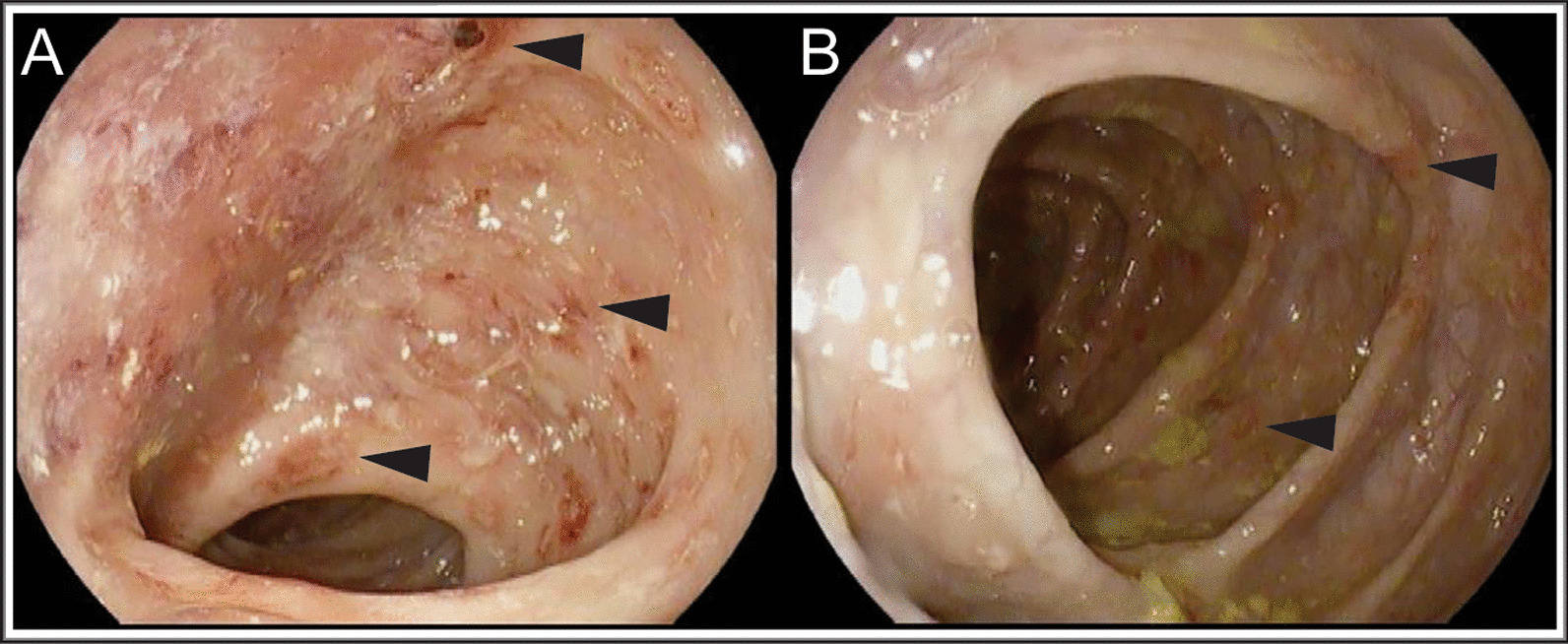
Gross intestinal pathology. Gross endoscopic image of the descending colon (**A**) and transverse colon (**B**) with evidence of diffuse erythema (black arrows), decreased vascular markings, and scattered erosions (black arrowheads)

He had a normal stooling pattern without hematochezia, making typical gastrointestinal infectious processes less likely, confirmed by negative stool culture and ova/parasite studies. He had further assessment for malabsorption as a reason for caloric losses, including qualitative fecal fat and stool reducing substances that returned normal. Although no gross features of his stool suggested intestinal inflammation or overt bleeding, given his persistent weight loss, systemic inflammation, and anemia, he was screened for IBD with fecal calprotectin. This returned markedly abnormal at > 1250 mg/g (normal, < 50 mg/g), which prompted further direct visualization with endoscopy. This revealed diffuse inflammation in the stomach, duodenum, and ileum, and throughout the colon (Fig. 3).

**Fig. 4:**
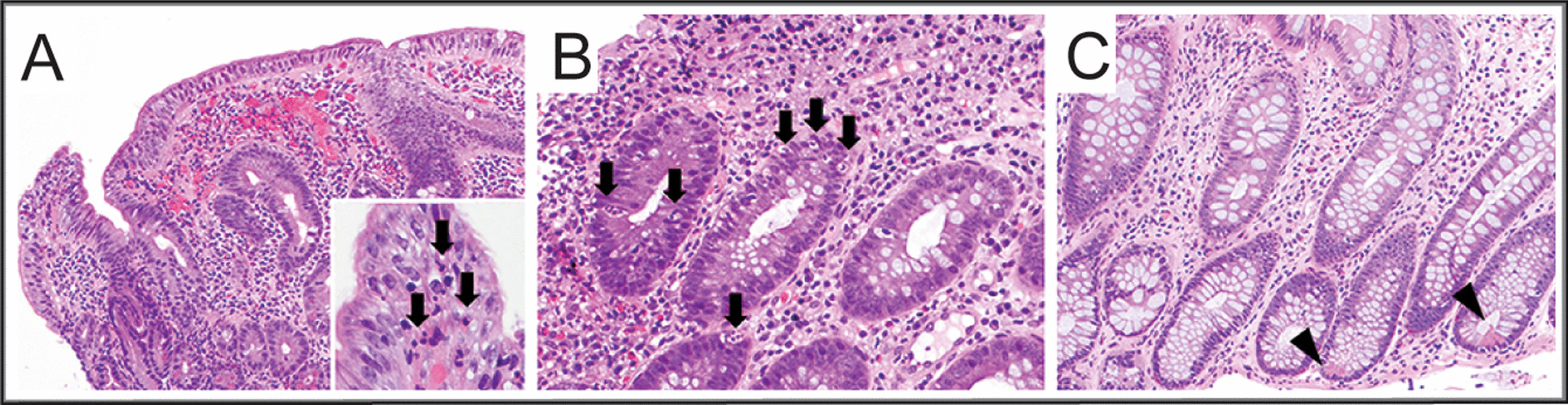
Intestinal histology. Duodenal biopsies (**A**) showed mild active inflammation and villous blunting (100×); inset is the villous tip with intraepithelial neutrophils (black arrows, 400×). Sigmoid colon biopsies (**B**) showed cryptitis with intraepithelial neutrophils (black arrows, 200×). Focal Paneth cell metaplasia (arrowheads) is identified in the descending colon (**C**, 100×), suggesting chronic inflammation. All images are from hematoxylin- and eosin-stained slides

Histologic examination of endoscopic biopsies (Fig. 4) revealed chronic inflammation in the stomach and acute inflammation in both the small and large intestines. Microscopic ulceration and cryptitis were seen throughout the intestines, and within the small intestines there was villitis associated with villous blunting; in sum, these features were consistent with IBD. Given his small bowel involvement, Crohn disease was favored over ulcerative colitis. He had been treated with scheduled nonsteroidal anti-inflammatory drugs (NSAIDs) for 5 days immediately prior to this endoscopy. Given that his weight loss predated this exposure, his NSAID exposure was brief, and his inflammation included both chronic features and was longitudinally extensive, NSAID enteropathy was judged to be a less likely cause. Similarly, his markedly elevated calprotectin level predated this NSAID exposure.

On synthesis of all relevant findings, including chronic bone marrow inflammation, systemic sequelae of inflammation (weight loss, fatigue, and chorea) in the absence of findings supporting a neoplastic, infectious, or toxic etiology, he was diagnosed with chronic nonbacterial osteitis (CNO) and inflammatory bowel disease. First-line treatment for CNO is scheduled NSAIDs, but NSAIDs are contraindicated with active colonic inflammation. Thus, his therapy was escalated to adalimumab, a monoclonal antibody against tumor necrosis factor-alpha (TNF-α), in an attempt to address both of his inflammatory conditions. After 6 months of twice-monthly adalimumab therapy, his pain and weakness had completely resolved, his weight resumed his prior growth trajectory at the 30th percentile, and his chorea had resolved. His labs and imaging also normalized, including prior bone marrow changes on whole-body MRI. Eighteen months following presentation, he remained in good health with no alternate diagnoses suggested.

## Discussion and conclusions

CNO is a rare autoinflammatory disorder affecting bone and other tissues that primarily affects children and adolescents. The classic presentation includes bony pain without significant systemic inflammation. CNO has features overlapping with other inflammatory and noninflammatory disorders, leading to a 15-month delay in diagnosis, on average [6]. Though it is noninfectious, CNO is speculated to be triggered by an infection that may induce immune dysregulation and impairment of pro-inflammatory downregulation, with subsequently increased presence of interleukin-1-beta (IL-1β) and TNF-α [7]. The differential diagnosis includes malignancy (leukemia, lymphoma, various types of histiocytosis or metastatic cancer) and infectious osteomyelitis (bacterial or fungal). The classic location of bony involvement is the clavicle, with general predilection for tubular bones, though any bone can be involved. Areas of involvement tend to be symmetrical and multifocal, most commonly within the metaphysis directly adjacent to the physis, as seen in this patient. There is a notable lack of surrounding inflammation, including soft tissue edema. [8]

There are various proposed diagnostic criteria for CNO, but none yet validated in children. The Bristol criteria (Table 1) may be the most commonly cited in the literature [6]. This patient met criteria for CNO with bone pain, typical MRI findings, and both criteria 1 and 2 (multifocal bone involvement and bone biopsy with inflammatory changes). Without full remission of bone pain between the first and second hospitalizations, this patient met criteria for CNO rather than the more severe chronic recurrent multifocal osteomyelitis (CRMO), which consists of episodic recurrences of inflammatory bone lesions and has a poorer prognosis.

**Table 1:** Bristol criteria for chronic nonbacterial osteitis

**The presence of typical clinical findings** (bone pain ± localized swelling without significant local or systemic features of inflammation or infection
AND
The presence of typical radiological findings (plain x-ray: showing combination of lytic areas, sclerosis, and new bone formation or preferably STIR MRI^a^: showing bone marrow edema ± bone expansion, lytic areas, and periosteal reaction
AND EITHER
**Criterion 1**: more than one bone (or clavicle alone) without significantly raised C-reactive protein (CRP^b^ < 30 g/L)
OR
**Criterion 2**: if unifocal disease (other than clavicle), or CRP > 30 g/L, with bone biopsy showing inflammatory changes (plasma cells, osteoclasts, fibrosis or sclerosis) with no bacterial growth while not on antibiotic therapy

^a^STIR MRI: short-tau inversion-recovery magnetic resonance imaging

^b^CRP: C-reactive protein

This patient’s presentation was unique with the presence of systemic inflammation, including weight loss (with caloric consumption greater than expected needs), fatigue, and markedly elevated ESR. Patients on the disease spectrum of CNO and CRMO can have varying degrees of systemic inflammation, which makes diagnosis challenging [9, 10]. However, weight loss is rarely reported with CNO/CRMO in isolation and should prompt further investigation for overlapping diagnoses, as in this case [11].

CNO has been reported as an extraintestinal manifestation (EIM) of IBD, and up to 30% of patients with inflammatory bowel disease can present with an EIM months to years prior to onset of inflammatory bowel symptoms [5, 12–14]. Importantly, this patient also had none of the classic symptoms of IBD, such as persistent diarrhea, gross hematochezia, or abdominal pain, suggesting early presentation of disease. Initial investigation for IBD was triggered by an elevated fecal calprotectin, a noninvasive screening test with high negative predictive value [15]. Endoscopically, he had diffuse erythema throughout his intestines, which correlated with microscopic evidence of active inflammation (ulceration, cryptitis, and villitis); however, he lacked microscopic evidence of the damage associated with chronic inflammation (crypt distortion, basal lymphoplasmacytosis, and granulomas) typically seen with IBD. Though he lacked the classic chronicity typically seen with IBD, the diagnosis of early IBD best encompassed his clinical picture: weight loss, systemic inflammation, endoscopic evidence of inflamed bowel, and finally presence of CNO, a rare but reported EIM of IBD.

Chorea can be a nonspecific feature of systemic inflammation, particularly in the presence of autoimmune diseases such as systemic lupus erythematosus and systemic vasculitis and/or the presence of antiphospholipid antibodies. Though Sydenham chorea is one of the major Jones criteria for diagnosis of ARF, this patient did not meet other criteria for diagnosis, and he did not have the typical accompanying emotional lability [16]. One prior report of transient chorea associated with IBD (ulcerative colitis), with normal brain imaging, has many parallels with our case [3]. Two other highly similar case reports are suggestive of this same association: one from 1979 and therefore lacking a modern immunological evaluation [17] and one detailed only in poster form [18].

The presence of chorea and weakness added to the extensive differential diagnoses for this patient. It was difficult to reconcile his presentation of systemic inflammation, weight loss, and extremity complaints with chorea into a unifying diagnosis. CNO rarely, if ever, presents with weakness; in this case, his weakness was thought to be secondary to pain from diffuse bony involvement, sciatic nerve irritation from local inflammation, or development of piriformis syndrome secondary to rapid weight loss [19]. Because of the chorea and weight loss, we also considered Huntington disease early in his evaluation, but the rapidity of his disease progression, the absence of neuropsychiatric symptoms or a suggestive family history, and his pain and weakness (not typical for Huntington disease) led us to discount this possibility. Finally, his normal brain MRI eliminated this diagnosis.

This patient’s ill appearance, with progressive cachexia despite caloric supplementation, created urgency to trial immunomodulatory therapy once infection had been ruled out. TNF-α has been implicated in the pathways for both CNO [20] and IBD [21], which simplified our choice for empiric anti-TNF-α therapy. The patient’s return to his asymptomatic baseline supported the role of systemic inflammation in his disease course, with components of both CNO, IBD, and inflammatory (transient) chorea all fully responding to immunomodulation.

CNO is a rare, diagnostically challenging disease that should be considered in a pediatric patient with unexplained systemic symptoms and bony pain. When combined with weight loss, CNO can be an extraintestinal manifestation of inflammatory bowel disease that may precede any intestinal symptoms. Neurologic manifestations—such as chorea—can also be a rare complication of systemic inflammatory disorders. The combination of musculoskeletal, neurologic, and systemic complaints in this child created a diagnostic dilemma that required extensive laboratory workup, multiple imaging modalities, histologic examination of areas of suspected involvement, and multidisciplinary collaboration to appropriately identify and treat the source(s) of systemic inflammation.

## Supplementary Information

**Additional file 1.** Physical exam maneuvers demonstrate motor impersistence, the hallmark feature of chorea. Note the intrusion of unwanted movements into limbs, face, and tongue when maintaining a fixed position.

## Data Availability

Not applicable.

## Abbreviations

IBD: Inflammatory bowel disease
CNO: Chronic nonbacterial osteitis
EIM: Extraintestinal manifestations
CRP: C-reactive protein
ESR: Erythrocyte sedimentation rate
MRI: Magnetic resonance imaging
PET–CT: Positron emission tomography–computed tomography
LCH: Langerhans cell histiocytosis
HLH: Hemophagocytic lymphohistiocytosis
ARF: Acute rheumatic fever
ECG: Electrocardiogram
NSAIDs: Nonsteroidal anti-inflammatory drugs
TNF-α: Tumor necrosis factor-alpha
IL-1β: Interleukin-1-beta
CRMO: Chronic recurrent multifocal osteomyelitis
